# Multilevel Coarsening for Interactive Visualization of Large Bipartite Networks

**DOI:** 10.3389/frma.2022.855165

**Published:** 2022-06-16

**Authors:** Alan Demétrius Baria Valejo, Renato Fabbri, Alneu de Andrade Lopes, Liang Zhao, Maria Cristina Ferreira de Oliveira

**Affiliations:** ^1^Department of Computing, Federal University of São Carlos, São Carlos, Brazil; ^2^Department of Computer Science, Institute of Mathematics and Computer Science, University of São Paulo, São Carlos, Brazil; ^3^Department of Computing and Mathematics, Faculdade de Filosofia Ciências e Letras de Ribeirão Preto (FFCLRP), University of São Paulo, Ribeirão Preto, Brazil

**Keywords:** complex network visualization, bipartite network visualization, exploratory network visualization, multilevel network visualization, multilevel network coarsening

## Abstract

Bipartite networks are pervasive in modeling real-world phenomena and play a fundamental role in graph theory. Interactive exploratory visualization of such networks is an important problem, and particularly challenging when handling large networks. In this paper we present results from an investigation on using a general multilevel method for this purpose. Multilevel methods on networks have been introduced as a general approach to increase scalability of community detection and other complex optimization algorithms. They employ graph coarsening algorithms to create a hierarchy of increasingly coarser (reduced) approximations of an original network. Multilevel coarsening has been applied, e.g., to the problem of drawing simple (“unipartite”) networks. We build on previous work that extended multilevel coarsening to bipartite graphs to propose a visualization interface that uses multilevel coarsening to compute a multi-resolution hierarchical representation of an input bipartite network. From this hierarchy, interactive node-link drawings are displayed following a genuine route of the “overview first, zoom and filter, details on demand” visual information seeking mantra. Analysts may depart from the coarsest representation and select nodes or sub-graphs to be expanded and shown at greater detail. Besides intuitive navigation of large-scale networks, this solution affords great flexibility, as users are free to select different coarsening strategies in different scenarios. We illustrate its potential with case studies involving real networks on distinct domains. The experimental analysis shows our strategy is effective to reveal topological structures, such as communities and holes, that may remain hidden in a conventional node-link layout. It is also useful to highlight connectivity patterns across the bipartite layers, as illustrated in an example that emphasizes the correlation between diseases and genes in genetic disorders, and in a study of a scientific collaboration network of authors and papers.

## 1. Introduction

Bipartite networks are a special type of network in which the set of nodes is split into two independent partitions called “layers” (they are hence called “two-layer” networks) and the links connect nodes from different layers. They emerge in important data mining problems that require modeling relations between two types of entities, e.g., documents and terms, papers, and authors (Newman, 2001; Grujić, 2008; Faleiros et al., 2017), or patients and genes (Hwang et al., 2008). Some authors even argue that many real-world unipartite networks are actually projections of bipartite networks (Guillaume and Latapy, 2004, 2006).

Exploratory visualization of network topology plays a relevant role in many data mining and optimization problems modeled as networks. Effectively conveying the relevant topological information of large-scale networks is challenging in interactive visualization (Tang et al., 2015; Staudt et al., 2016), and even more so in handling bipartite networks, in view of their peculiar organization and inherent topological complexity. A few recent contributions discuss strategies for interactive visualization of such networks (Chan et al., 2018; Garcia-Algarra et al., 2018; Pezzotti et al., 2018; Steinbock et al., 2018; Sun et al., 2019; Zhao et al., 2019; Waldner et al., 2020), as further discussed in Section 3.

Analysis assisted by interactive visualization is arduous because human perceptual abilities are limited to rather small network sizes even when executing simple tasks such as locating a node or finding the links between a pair of nodes (Ghoniem et al., 2005). As network sizes increase, even simple data exploration tasks become increasingly difficult. Furthermore, besides the cognitive burden on users attempting to grasp both local and global information, a heavy rendering load impairs smooth navigation on node-link views (Ghoniem et al., 2005). Limitations of node-link representations encouraged many research efforts on devising alternative visualization techniques, with relative success (Nobre et al., 2020; Di Giacomo et al., 2021). Yet, node-link views remain a classic approach for interactive visualization of networks, even if to be used in combination with other techniques (Di Giacomo et al., 2021).

In order to reduce clutter and obtain representations more amenable to user interaction, general-purpose interactive visualization of large unipartite networks often relies on node aggregation into clusters or communities (Abello et al., 2006; Archambault et al., 2008; Wong et al., 2009; Batagelj et al., 2010; Dias et al., 2017b; Perrot and Auber, 2018) (see Section 3). Nevertheless, a single level partition of the set of nodes may prove ineffective, as important topological structures often manifest themselves at multiple observation levels. Indeed, alternative solutions have been introduced that rely on hierarchical algorithms for clustering or community detection to obtain a hierarchical aggregation to support user navigation and exploratory tasks. Thus, it is possible to interact with higher-level representations in order to make early assessments before proceeding to detailed investigation. This strategy is likely to be more effective than continuously rendering the full network in situations where the user focus is directed to a specific region. A hierarchical network model offers a natural support to the well-known “visual information seeking mantra” of information visualization (Shneiderman, 1996), where users depart from an overview and then navigate and interact in the visual space to focus on target regions and demand further details. This mantra embeds a number of visual design guidelines and is widely acknowledged as a general interaction framework in designing information visualization applications.

Alternatively, a hierarchical aggregation of a complex network may be obtained with the multilevel method, a meta-heuristic employed to modify and potentially fix a solution obtained with a target optimization algorithm (Karypis and Kumar, 1998; Wong et al., 2009). A multilevel algorithm performs an incremental coarsening of an original network to yield a hierarchy of simplified networks from the original (Brandt, 1988; Karypis and Kumar, 1998). It yields a hierarchical representation, where each level depicts a network with fewer nodes and links than its previous one. Multilevel methods have been originally introduced to enable executing expensive algorithms on large-scale networks.^1^ The rationale is to compute an initial solution executing a target algorithm on a simpler network that still preserves the relevant properties of the original. This initial solution is then incrementally refined over the inverse hierarchy of coarsened networks to yield an approximate solution to the original problem (Noack and Rotta, 2009; Valejo et al., 2018). Applying the strategy successfully required two essential capabilities, namely finding a coarsest network that preserves the essential features of the original, and generalizing the initial solution obtained to the full original model. Thus, identifying a coarsening strategy that yields a suitable trade-off between solution accuracy, generalization capability, and speed is critical. This is commonly approached with empirical investigation on each problem and data set, sometimes departing from existing coarsening algorithms, sometimes introducing novel ones. Yet, multilevel methods have been successfully employed in a range of important problems defined in unipartite networks (Brandt, 1988). It has also been considered to accelerate the computation of node-link layouts for purposes of network drawing and visualization (Harel and Koren, 2000a; Walshaw, 2000; Hachul and Jünger, 2004; Hu, 2005; Archambault et al., 2007; Frishman and Tal, 2007; Bartel et al., 2010; Arleo et al., 2016; Toosi and Nikolov, 2016; Hinge et al., 2017).

In earlier work, authors of this paper introduced a general multilevel framework for bipartite networks, i.e., it preserves the bipartite restrictions and ensures efficient coarsening in this context (Valejo et al., 2017a, 2018, 2020a). The solution was motivated by the growing occurrence of bipartite networks as models for solving important real-world problems, e.g., the method has been applied in problems of link prediction (Ferreira et al., 2019), trajectory mining (Minatel et al., 2018, 2019), and community detection (Valejo et al., 2014a,b, 2020a).

In this contribution, we address the problem of employing the multilevel method for interactive visualization of large bipartite networks. As multilevel network coarsening can preserve the topological structures relevant to problem solving, we propose using coarsening algorithms to obtain multi-resolution networks, which can be rendered as node-link diagrams at distinct levels of detail for purposes of interactive visualization. To the best of our knowledge, multilevel coarsening has not been considered so far for interactive visualization of real-world bipartite networks (Valejo et al., 2020b). We follow this premise and introduce a proof-of-concept solution that supports user-driven navigation in a hierarchical multilevel representation. The distinctive points of our contribution are the following:

1. We define a visualization strategy on top of an existing general multilevel framework for bipartite networks. Our strategy allows plugging-in any network coarsening algorithm, yielding maximum flexibility in obtaining multi-resolution representations of a given network. Thus, given an input network, alternative multi-resolution models can be created based on user convenience and her choice of coarsening algorithm.
2. We use our strategy as the underlying model of a system that supports interactive visualization and navigation on the multi-resolution network models, starting with a node-link view of the coarsest network. User interaction triggers gradual, on-demand expansion or contraction of selected sub-sets nodes. Thus, at any moment a node link view may be rendered with different regions shown at distinct resolution levels. The system also offers functionalities for zooming in/out and requesting complementary information on nodes or links.
3. The system is introduced as a proof-of-concept to demonstrate the feasibility of the strategy to support effective exploratory visualization of complex bipartite networks. The operation of the strategy and its potential are illustrated with examples in which real-world bipartite networks with varied topological properties are explored on-demand.

The remainder of this paper is organized as follows: in Section 2 we present a brief overview of our previously introduced multilevel framework for bipartite networks, which provides the underlying framework for the proposed visualization strategy. In Section 3, we briefly review related work on interactive network visualization, with an emphasis on solutions aimed at bipartite networks. In Section 4, we describe the proposed visualization strategy built on top of the general multilevel method, and describe how it has been implemented into a proof-of-concept system. In Section 5, we report illustrative case studies using the strategy in different scenarios. Finally, in Section 6, we summarize our findings and discuss future work.

## 2. Multilevel Method on Bipartite Networks

**Figure 1:**
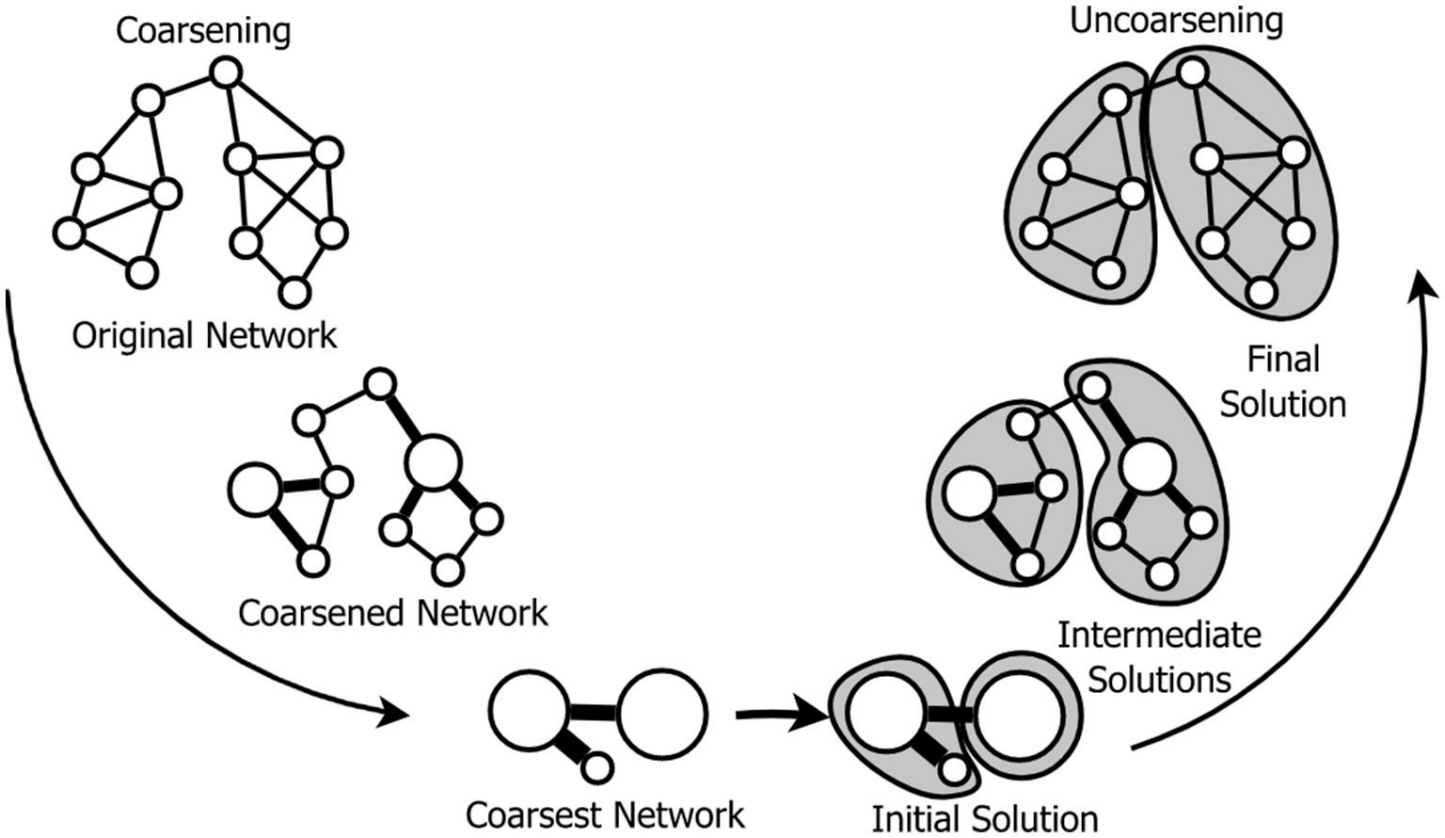
Phases of a general multilevel optimization method operating on a network. (i) Coarsening: computes a sequence of incrementally simpler networks. (ii) Solution finding: computes an initial solution executing a target algorithm in the coarsest network. (iii) Uncoarsening: projects this solution back up to the original network.

The overall strategy of a multilevel method in the context of solving problems in networks is illustrated in Figure 1. The method operates in three phases, identified as coarsening, solution finding and uncoarsening. A description of the phases requires establishing some definitions and notation, as follows.

A unipartite network is represented by G=(V,E,σ,ω), wherein V and E define the set of nodes and links, respectively, and a link (v,u)={(u,v)=(v,u)∣u,v∈V}. n=|V| and m=|E| denote the number of nodes and links, respectively. A link (*u, v*) or a node *u* can associated with a weight, denoted, respectively, as $\omega \left(u,v\right):V\times V\to {\mathbb{R}}^{*}$ or $\sigma \left(u\right):V\to {\mathbb{R}}^{*}$. The network is bipartite if V is split into two sub sets $V^{1}$ and $V^{2}$, such that $V^{1}\cap V^{2}=\emptyset$ and $E\subseteq V^{1}\times V^{2}$.

The *h*-hop neighborhood of a node *u*, denoted by Γ_*h*_(*u*), is given by the nodes distant from *u* by *h* or less links. Thus, Γ_1_(*u*) is the set of nodes adjacent to *u*; Γ_2_(*u*) is the set of nodes 2-hops away from *u*, and so forth. The degree of *u*, denoted by κ(*u*), is the number of its incident links, i.e., |Γ_1_(*u*)|. A similarity measure (henceforth called similarity *index*) is a function that quantifies common properties of a pair of nodes (*u, v*), yielding values (scores) in the range [0, 1]⊂ℝ, from lowest (0) to highest (1) similarity (Valejo et al., 2014a). A typical structural index is the number of common neighbors, defined as *CN*(*u, v*) = |Λ(*u, v*)|, wherein Λ(*u, v*) = {Γ_1_(*u*)∩Γ_1_(*v*)}.

Back to the multilevel method, in the coarsening phase a given network $G_{0}$ is iteratively coarsened into a hierarchy of increasingly simpler representations, yielding a multilevel hierarchy $\left\{G_{1},G_{2},\cdots \;,G_{H}\right\}$ with H levels. The hierarchy describes $G_{0}$ at multiple levels-of-detail, wherein $G_{H}$ is the *coarsest network*. Specifically, a coarsening algorithm constructs a coarser network by collapsing a set of matched nodes {*u*_1_, …, *u*_*n*_} into a single *super-node* *sV*_*i*_. The nodes in set $\left\{u_{1},\ldots ,u_{n}\right\}\in V_{h}$ collapsed into a super-node *sV*_*i*_∈*V*_*h*+1_ are called *predecessors* of *sV*_*i*_, denoted Θ(*sV*_*i*_), whilst the super-node (*sV*_*i*_) is a *successor* of its originating nodes, denoted Δ(*u*_*i*_). A successor network $G_{h+1}$ will inherit the non-matched nodes from its predecessor network *G*_*h*_. In order for *G*_*h*+1_ to be a good proxy to its predecessor network, the weight σ(*sV*_*i*_) of a super-node *sV*_*i*_∈*V*_*h*+1_ is computed as the sum of weights of its predecessor nodes. Furthermore, any links incident to nodes in Θ(*sV*_*i*_) are collapsed into the so-called *super-links* incident to *sV*_*i*_.

In the solution finding phase, a target task, such as community detection, dimension reduction, link prediction or node-link layout computation, is evaluated in $G_{H}$ yielding an initial solution $S_{H}$. In the uncoarsening phase $S_{H}$ is gradually refined through the intermediate networks $G_{H-1},G_{H-2},\ldots ,G_{1}$ up to the original network $G_{0}$.

Notably, effective application of the strategy to any problem is highly dependent on the quality of the hierarchical multilevel representation built. Coarsening requires a policy to collapse nodes and links into super-nodes and super-links; uncoarsening requires a policy to expand collapsed nodes or links. Clearly, the steps of computing an initial solution and refining it are task dependent. As opposite, policies for merging nodes and links may be defined in terms of the network topology only, regardless of the task (Valejo et al., 2020b). Here we briefly introduce a few coarsening strategies for bipartite networks defined in earlier work, namely algorithms *OPM*, *RGMb*, *GMb*, and *MLPb*.

*OPM* (one-mode projection-based matching algorithm) is one of the earliest coarsening algorithms applied to bipartite networks (Valejo et al., 2017a,b). It decomposes the bipartite network G into two unipartite networks, one relative to each layer, i.e., $G^{1}$ and $G^{2}$, which allows employing solutions devised for unipartite networks, such as the popular Heavy-Edge Matching (*HEM*) algorithm (Karypis and Kumar, 1998). In *HEM*, a random node *u* is matched with an adjacent node *v* for which the edge (*u, v*) has maximum weight overall all edges adjacent to *u*.

Valejo et al. (2018) designed two coarsening algorithms specifically for bipartite networks. Algorithms *RGMb* (Random greedy matching) and *GMb* (Greedy matching) extend earlier link-based matching strategies which operate by collapsing node pairs into a single super node. Both inspect the 2-hop neighborhood of a node *u* to identify matching candidates, picking the node with the greatest similarity index with *u*. *RGMb* picks candidate nodes for matching randomly, whereas *GMb* picks nodes from a priority queue holding the most similar pairs.

A third algorithm called *MLPb* (Multilevel label propagation for bipartite networks) (Valejo et al., 2020a), has been introduced based on the well-known *Label Propagation Algorithm* (*LPA*) (Raghavan et al., 2007). Each node is initially assigned a unique label, and at each iteration node labels are updated with the node's most frequent label in its two-hop neighborhood. Groups of nodes with the same label are collapsed into a single super-node. Coarsening with *MLPb* typically requires less iteration steps than RGMb or GMb, as unlike these the algorithm is not restricted to collapsing just a pair of nodes per iteration. Preliminary studies provided empirical evidence that multilevel representations obtained with *MLPb* preserve the essential topological features of a bipartite network (Valejo et al., 2020a).

Of course, the choice of coarsening algorithm affects the multilevel representation obtained, which is key to the interactive visualization process. There is no better algorithm *a priori*, as the suitability of a representation must be considered in the context of the user problem, tasks and goals. This is a problem that deserves further investigation. Interestingly, the impact of distinct coarsening policies on the effectiveness of the multilevel strategy applied in different data mining tasks is also a relevant research topic where interactive visualization itself can play an important role (Valejo et al., 2021).

## 3. Interactive Visualization of Large Network

Whilst node-link representations are intuitive to convey network topology, they have very limited scalability (Ghoniem et al., 2004; Nobre et al., 2020; Di Giacomo et al., 2021). Rendering large networks can be slow and the result can be cumbersome. For unipartite networks it is common to use aggregations of nodes and/or links to mitigate clutter and reduce rendering load. Link bundling is possibly the best established solution for implicit link aggregation (Holten, 2006; van der Zwan et al., 2016; Lhuillier et al., 2017), whereas node aggregation strategies are usually explicit and domain-driven, with a diversity of strategies reported in the literature. In general terms, most solutions use clustering or community detection algorithms to group nodes into meta-nodes, super-nodes, clusters or communities that may be handled as individual entities (Von Landesberger et al., 2011), yielding a hierarchical representation that can be navigated and explored for visualization and analysis purposes. Hierarchical algorithms have been employed to create simplified representations of large networks (Dias et al., 2017a), and multilevel methods have been applied, for instance, to reduce the computational cost of computing node-link layouts (Harel and Koren, 2000b; Gajer and Kobourov, 2001; Hachul and Jünger, 2004; Wong et al., 2009). An experimental evaluation has been reported on using multilevel algorithms in association with energy-based layout algorithms (Bartel et al., 2011).

Still on the realm of unipartite networks, Abello et al. (2006) uses clustering algorithms to define a hierarchy over the network, so that users can navigate in a top-down strategy by interactively expanding individual clusters. Archambault et al. (2008) proposes using domain-specific attributes associated with the nodes and edges to create different possible hierarchies on the same unipartite network, instead of a single, fixed hierarchy as obtained with clustering. Wong et al. (2009) introduces a coarsening algorithm to create a sequence of reduced networks that retain the most important structural features at each level. A coarser graph is guaranteed to have no less than half the number of nodes than the previous (less coarsened) graph, i.e., ensuring a 50% reduction rate at each level. Similarly, Dias et al. (2017b) uses a non-negative matrix factorization (NMF) to rewrite the edge weights and then employ a coarsening algorithm to create a sequence of simpler networks. Batagelj et al. (2010) introduced a two-level clustering algorithm that guarantees the intra and the inter-cluster edges satisfy a set of desired topological properties. This strategy makes it possible to exploit and combine different visualization algorithms. Perrot and Auber (2018) presents a technique to visualize huge graphs using a client running in a Web browser. For this purpose, the authors introduce novel scalable algorithms based on the well-known *k*-means clustering to produce multiple levels of abstraction of the network.

Another category of related work comprises solutions introduced for exploratory visualization of data modeled as large scale bipartite graphs, which are becoming more prevalent recently. Several systems employ the biclustering algorithm (Heinrich et al., 2011) to generate node aggregations in this context (Dörk et al., 2012; Xu et al., 2016; Steinbock et al., 2018; Zhao et al., 2018; Sun et al., 2019). Using a co-clustering algorithm, the interactive visualization system by Xu et al. (2016) assists user recommendation on cluster models to be input into a machine learning algorithm (Hoi et al., 2010) that will learn acceptable clusterings according to user preferences. Chan et al. (2019) also use co-clustering to facilitate interactive data exploration. Waldner et al. (2020) deal with interactive exploration of time-dependent large bipartite graphs. They use two clustering algorithms to build a hierarchical aggregation: a biclustering algorithm to group nodes so as to maximize the graph modularity, and a time series clustering algorithm to group nodes based on their temporal correlation. The visual encoding adopts the usual approach of presenting two vertical lists of nodes, laid out in parallel. Other approaches are also employed to assist visualization of bipartite graphs, as in Garcia-Algarra et al. (2018), who use a k-core decomposition to identify and aggregate groups of nodes that share connectivity properties in order to simplify the network structure. The rationale of aggregating groups of nodes that share connectivity properties is also at the core of multilevel coarsening, employed in this paper.

Yet, other approaches are possible. The system WAOW-Vis (Pezzotti et al., 2018), for instance, adopts a hierarchical dimensionality reduction technique to create hierarchical representations of bipartite graphs. Users may interact to expand nodes in a particular area. The BiCFlows system (Steinbock et al., 2018) relies a novel visualization to support exploration of large weighted bipartite graphs, using biclustering for a hierarchical aggregation. Selecting a clustering yields a new execution of the biclustering algorithm to support a more detailed visualization. The ViBr tool (Chan et al., 2019) uses the Minimum Description Length (MDL) principle to create a clustered network, which can be explored selecting specific clusters and re-executing the MDL approach. The system employs adjacency lists for visualization, rather than node-link views. Zhao et al. (2019) also prefer to adopt an alternative representation to the node-link view, in this case a matrix-based visualization to assist in interactive analysis of missing links in bipartite networks.

Regarding multilevel methods in association with visualization of bipartite networks, very few specific strategies have been designed. Cintra et al. (2019) introduces a visualization metaphor in association with the multilevel method, however the focus is not on exhibiting network topology, but in assisting developers of multilevel methods to compare the outcomes of different coarsening algorithms. As opposite, Valejo et al. (2020a) illustrate how the *MLPb* coarsening algorithm can support node-link visualizations of large bipartite networks, however the authors only consider static visualizations, rather than interactive exploratory visualization scenarios.

In this work, we contribute a novel approach for interacting with node-link views of large bipartite networks. Navigation of the network at varying levels-of-detail is afforded by a multi resolution hierarchical representation of the network obtained with multilevel coarsening algorithms. We are not aware of previous efforts of using multilevel network coarsening in this context.

## 4. Multilevel Visualization of Bipartite Networks

In this section, we describe how a multi-resolution network representation $\left\{G_{1},G_{2},\cdots \;,G_{H}\right\}$ obtained with a multilevel coarsening algorithm can provide an underlying hierarchical representation for interactive exploration of an original large-scale bipartite network $G_{0}$. We specialize the general framework depicted in Figure 1 to address the target task of constructing a multiresolution visual mapping of $G_{0}$. In this context, the initial solution $S_{H}$ corresponds to any suitable node-link layout of its coarsest representation $G_{H}$. However, rather than pre computing a layout for each intermediate network $G_{h}$, we introduce a strategy that performs uncoarsening locally over sub-graphs defined by user selections. In other words, whenever the user wishes a more detailed view of a certain region of the graph than the one currently displayed, s/he identifies and selects the corresponding super-nodes and the uncoarsening algorithm is triggered on demand, on the selected super-nodes. The opposite is also possible, i.e., changing from a detailed current view of a sub-set of nodes to a coarser view by collapsing a selected sub-set of nodes into super-nodes.

As further detailed in Section 4.2, expanding selected super-nodes for a more detailed view requires identifying their predecessor nodes at the upper hierarchical level, which must be incorporated into the rendering layout. Likewise, collapsing user selected nodes requires identifying their successor super-nodes, then updating the rendering accordingly. This solution prevents unnecessary complexity and, most importantly, avoids overloading the visualization with information beyond the cognitive convenience of the user and the computational power available to ensure real-time interactivity. On the other hand, the node-link layout of a network displayed at any moment may be depicted with different regions shown at distinct resolution levels, following the user interactions.

**Figure 2:**
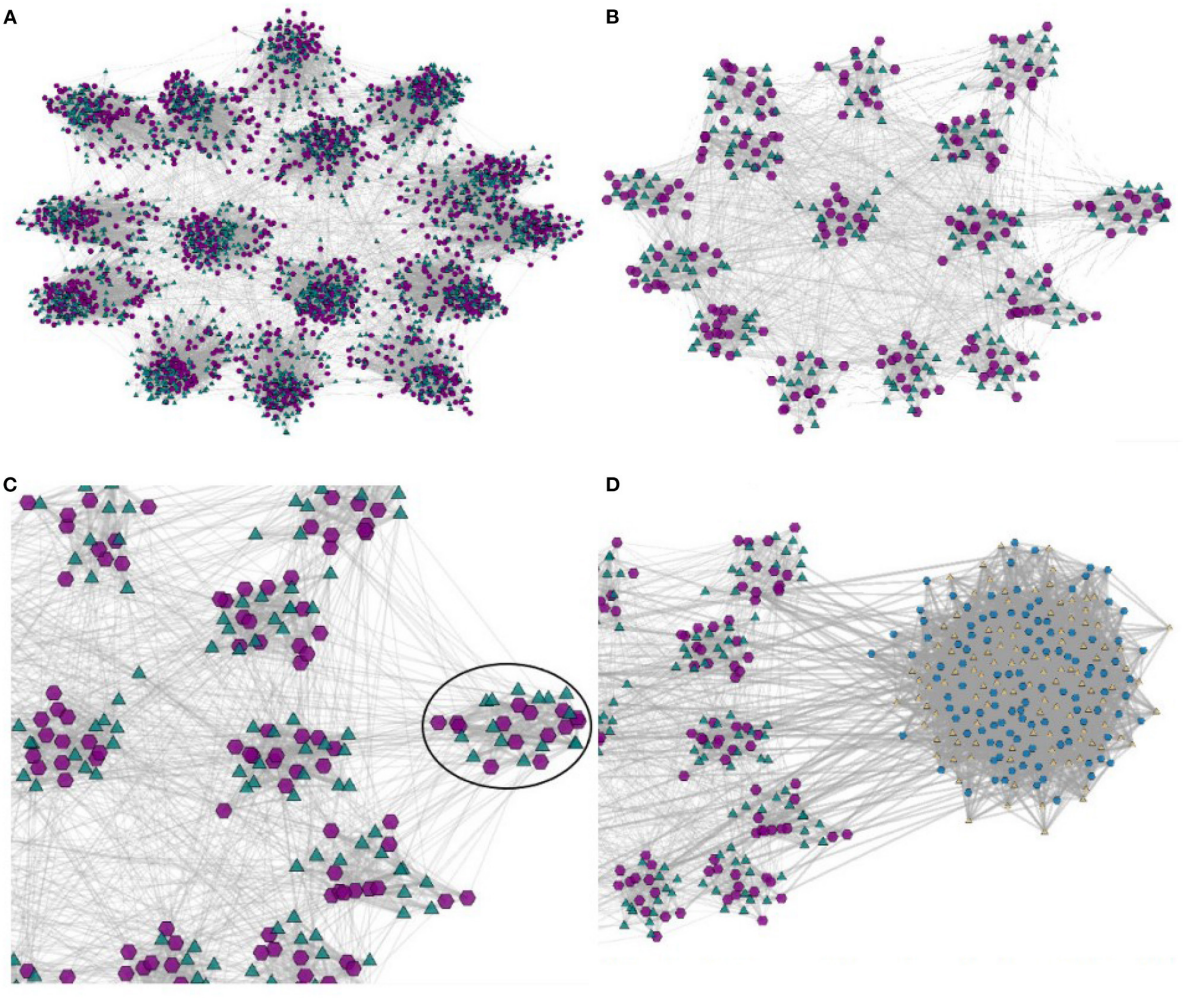
The navigation pathway for exploring a bipartite network made possible by coupling appropriate interaction functions with a hierarchical model obtained applying multilevel coarsening to the original network. The bottom level of the hierarchy corresponds to the full network, the upper level corresponds to its coarsest version, with intermediate networks in between. Compliant with the “visual information seeking mantra” (Shneiderman, 1996), it displays an initial high-level view of the coarsest network. A user may then expand selected nodes for a more detailed view of the corresponding local structure, which is achieved moving upwards in the hierarchy. S/he may also apply filters and modify the visual mappings of the topological elements, or again drill-down for further detail. S/he may also move downwards in the hierarchy, returning to a coarser view. These and other user operations may be carried out in any sequence. **(A)** Initial bipartite network. **(B)** Highest-level view (coarsest network). **(C)** Selecting a region in the coarsest view. **(D)** Detailed view of the selected nodes (in the black circle).

The approach is defined as a general framework that is independent of specific choices of either coarsening algorithms or node-layout algorithms; an implementation may thus offer multiple choices of such algorithms to meet distinct domain and/or user specific demands. Additionally, it seamlessly supports the “visual information-seeking mantra” (Shneiderman, 1996) of *overview first, zoom and filter, then details-on-demand*. Exploration starts with the user observing a node-link layout of the highest level network (its coarsest representation $G_{H}$), rendered with any efficient algorithm. S/he may interact to inspect topology, nodes or associated content, and eventually select an arbitrary sub-set of nodes to obtain an expanded view. In other words, the user is able to zoom into the region defined by the selected nodes, which are rendered at the next level of the hierarchical multilevel network $\left\{G_{0},G_{1},\cdots \;,G_{H-1}\right\}$, as illustrated in Figure 2. In the example, the initial node-link layout exhibits $G_{0}$, after a user interaction the layout is updated so that the selection is rendered at the level of detail given by $G_{1}$. Similar interactions can be applied to arbitrary regions of the network rendered at any level of detail captured by the multilevel representation, therefore allowing a user to switch between coarser to finer levels of detail, or vice-versa.

We named our approach BiNetVis (from Multilevel Bipartite Network Visualization), and it is described in Algorithm 1. The algorithm instantiates the general multilevel framework depicted in Figure 1 to the specific problem of multilevel visualization of bipartite networks. Initially, an input network $G_{0}$ is coarsened with an arbitrary user selected algorithm, indicated by the routine *Coarsen* (lines 1-3). Coarsening is performed independently on each network layer {1, 2}, or the user may choose to coarsen just one of the layers. The number of levels of the resulting network hierarchy, which defines the extent of the desired simplification, is typically a parameter of the coarsening algorithm. Next, any choice of node-link layout algorithm can be employed to render $G_{H}$ to the screen, line 4. For purposes of interactive exploratory visualization, network $G_{H}$, which depicts the coarsest representation of $G_{0}$, provides the initial focus for the user to interact. S/he can move the focus up or down the multilevel hierarchy while interacting, and the visual mapping is adjusted accordingly to the current focus. Possible user operations in the interaction loop (lines 5–15) would be requests to expand selected (super-)nodes or to collapse selected (super-)nodes (routines Expand and Collapse in lines 10 and 12, respectively). Although not explicit in the algorithm, additional operations can be incorporated into an implementation, e.g., to request metadata relative to nodes or links, modify graphical properties such as position, color or transparency of nodes and/or links; apply zooming or panning to the rendering, etc.

**Algorithm 1:**
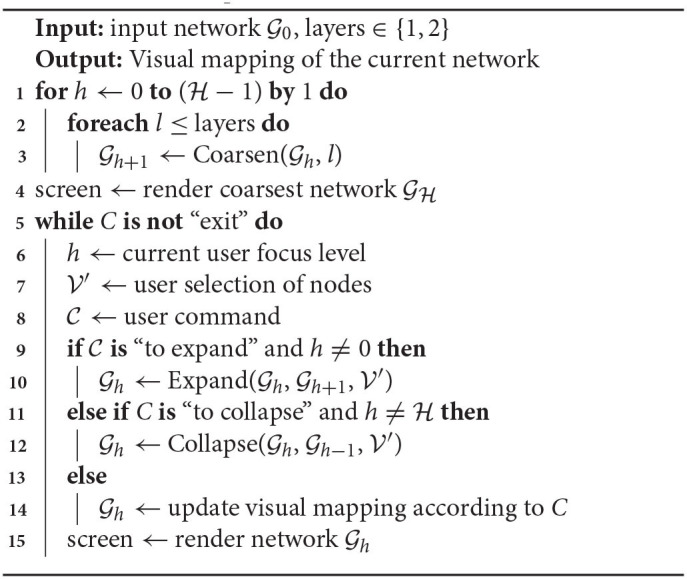
BiNetVis algorithm for multilevel visualization of a bipartite network.

Algorithms 2, 3 detail the routines *Expand* and *Collapse*, which take as inputs two networks $G_{h}$ and $G_{h+1}$ (or $G_{h}$ and $G_{h-1}$) where *h* is the level of the current viewing focus, and a user selection of nodes $V^{\prime }\in V_{h}$. Recall that set Θ(*u*) refers to the predecessor nodes of *u* at level *h*−1, and Δ(*u*) refers to the successor super-node of *u* at level *h*+1. Expanding a selection $V^{\prime }$ requires incorporating into the current network layout the predecessor nodes, at level *h*+1, of all nodes $v\in V_{h}^{\prime }$ and their adjacent links. Similarly, collapsing $V^{\prime }$ implies in replacing its nodes with their corresponding successor nodes and adjacent links at level *h*−1. In both cases, the node-layout rendering will be updated with a hybrid network model which simultaneously includes nodes and links from networks at multiple levels of the hierarchical multilevel representation.

**Algorithm 2:**
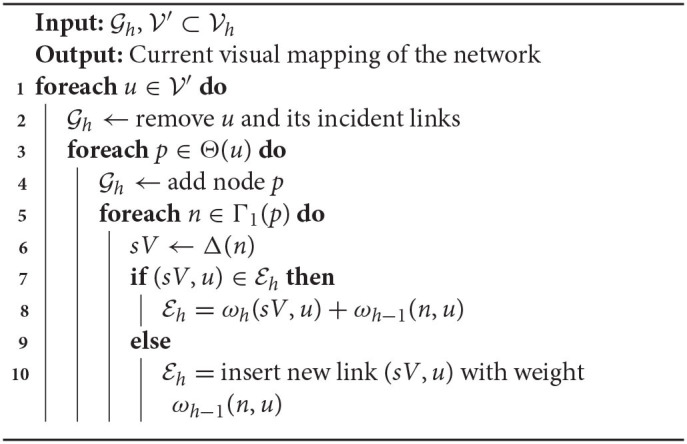
Expand selected nodes one level up.

**Algorithm 3:**
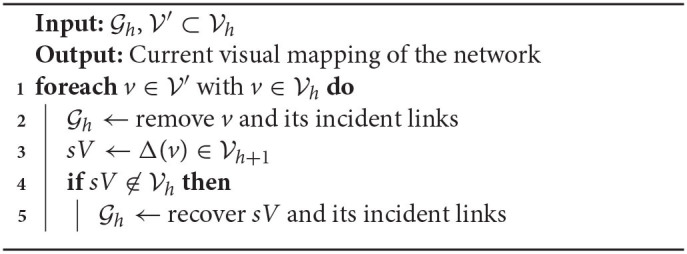
Collapse selected nodes one level down.

### 4.1. BiNetVis Interface

**Figure 3:**
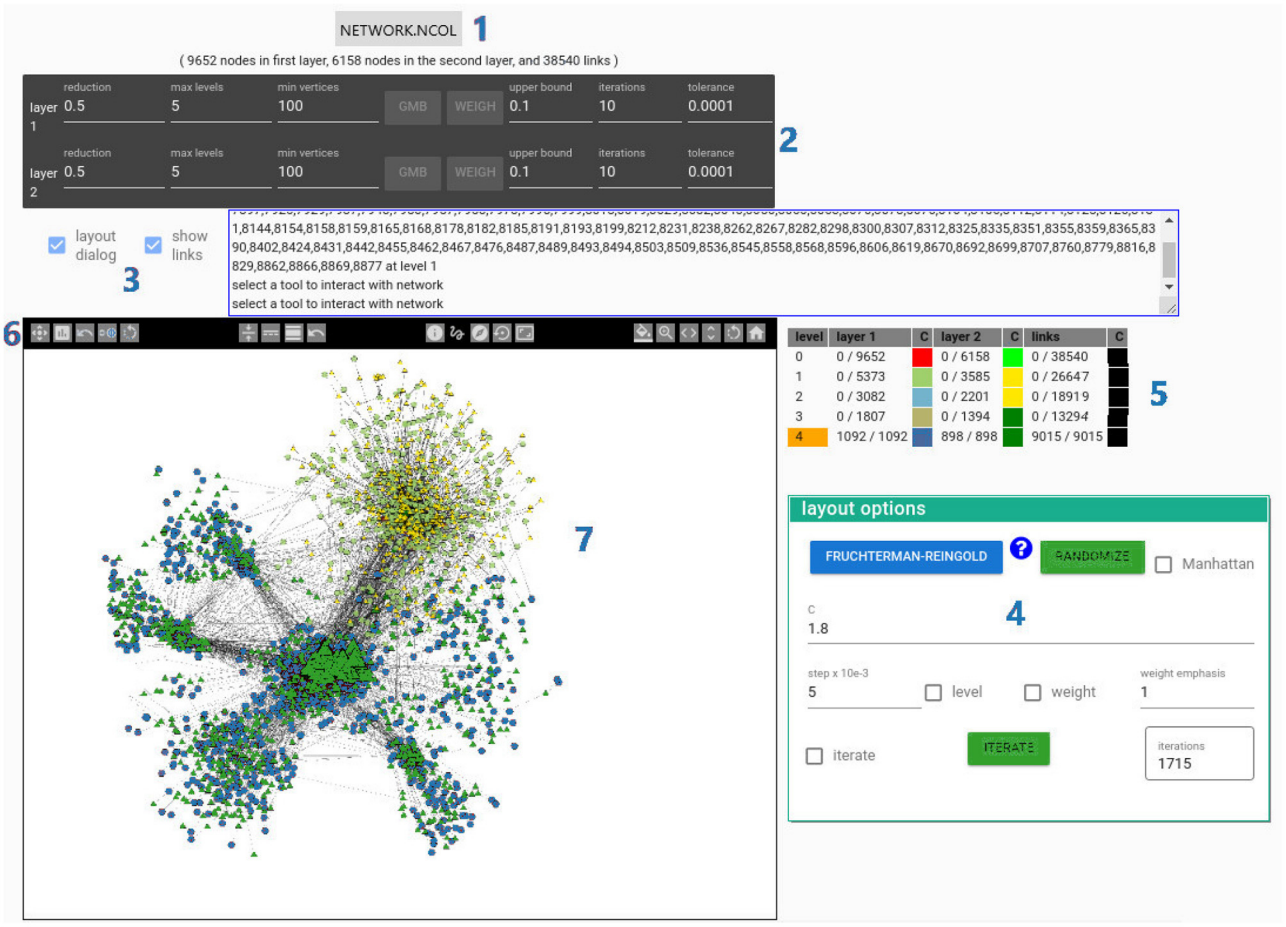
The BiNetVis system interface: (1) drop-down menu to select a network or upload a network for analysis; (2) control widgets of the coarsening algorithm and its parameters; (3) check boxes to display the layout dialog and show/hide links and area to display user requested information on nodes or links; (4) control widget of the node-link layout algorithm; (5) interactive table panel depicting the multilevel hierarchy levels with corresponding bipartite layers; also used to set the current hierarchy view focus for interaction on the canvas; (6) toolbar with controls for network navigation and visualization; (7) canvas with the hybrid node-link network view. This view shows nodes at two distinct levels of detail, indicated by the glyph colors: level 4 (blue/green) and level 1 (light green/yellow). The current view focus is set at level 4, as indicated by the orange mark at the corresponding entry in the multilevel table panel (5).

We developed a proof-of-concept implementation of the BiNetVis approach^2^; the system interface is illustrated in Figure 3. An analyst initially selects a network model from a drop-down menu (1), which also admits uploading a new network. BiNetVis currently admits networks described in the NCOL^3^ readable file format, often associated with biological data graphs, though other formats could be incorporated.

The next step once a network has been loaded is to select a coarsening algorithm and set its parameters in the corresponding menu (area 2 in Figure 3). The user can choose from OPM, GMb, RGMb, and MLPb coarsening algorithms (introduced in Section 2), and set their parameters—reasonable default settings are provided to newcomers. The options indicated in area 3 can be checked to hide/show the “layout options” menu and to hide/show edges in the layout rendering, in addition to showing the predecessor nodes of a selected super-node. Once the “render network” button is hit (area 4) the coarsening algorithm is activated to build the hierarchical representation, and a node-link layout of the coarsest network is computed with the selected layout algorithm and rendered to the canvas (area 7). The system currently incorporates two node layout algorithms, namely Fruchterman and Reingold (1991) and Kamada and Kawai (1989). The most relevant interface components to support user interaction in BiNetVis are the multilevel table panel (area 5) and the toolbar (area 6).

The table panel (5) provides the necessary controls to navigate in the multilevel hierarchy. The color pairs used in rendering the nodes change to identify the different levels, as indicated in the table panel. Because different regions of the network may be rendered at distinct levels of detail, a user must explicitly inform the view focus level *h* to which level the current operations apply. The default focus level is the coarsest one, and it may be changed by the user clicking in any line of the table panel that corresponds to the desired level focus. The current focus is signaled by the orange marker in the corresponding line, e.g., in Figure 3 the marker appears at the line corresponding to level *h* = 4, which is the coarsest representation of the input network. For each layer of *G*_*i*_ at level *h* = *i* the table panel also informs how many nodes and links are currently visible, vs. their total numbers. The user also has options to modify node colors or shapes, as well as to toggle on/off drawing of links by means of left/right clicks on the corresponding colored square. Any current user actions apply only to the nodes/links at the currently defined view focus level.

Interaction is governed by the controls in the toolbar (6), which are organized in four groups according to their target element (node, link, multilevel hierarchy, canvas). The leftmost group of controls affect the mapping of node properties to graphical attributes of its corresponding graphical marker. Moving right, the next group of controls affect the mapping of link properties to graphical attributes of the corresponding lines. The next group is of controls to navigate in the multilevel hierarchy, e.g., obtain information relative to a selected (clicked) node, its predecessors and successor, move nodes in the canvas by clicking and dragging; define a rectangular region in the canvas and drag to move all nodes within, expand super-nodes to show their predecessor nodes and links at a finer level of detail, or collapse super-nodes that have been previously expanded. Finally, the rightmost set of controls affect the canvas, with tools to change the background color and perform zooming, panning and rotation of the network.

### 4.2. BiNetVis Implementation

Components of the BiNetVis software are mostly written in a combination of JavaScript and Python: it uses Vue.js (set up by Nuxt.js) in the front-end client, the back-end is a Flask Python server, used to perform specialized or heavy calculations. A secondary server, a FeatherJS, is used to facilitate contact with the database and real-time multi-user interaction. The data is stored in a MongoDB database and ordinarily in the file system, while the multi-user interaction is currently deactivated to avoid unnecessary complexity. Multiple bipartite network coarsening algorithms available from a previous implementation (Valejo et al., 2018) are accessed by the Flask server. Pixi.js is employed for fast WebGL 2D rendering on the canvas. The choice of features and technologies supports visualization of networks with tens of thousands of nodes without any perceptible lag in the interactive navigation and transformations, with links rendered, even when running the system on ordinary machines, e.g., with 8GB RAM DDR3, a first generation i7 processor and a 1GB GPU.

In order to comply with the goal of handling large bipartite networks, the glyphs on the canvas use only triangles as geometric primitives (for nodes) and straight line segments (for links). It is possible to choose the colors and shapes associated with the bipartite layers at the different hierarchy levels, and also to alleviate the computational burden by setting the option of not rendering the links.

## 5. Use Case Scenarios

We present four use case scenarios to illustrate the flexibility afforded by the proposed visualization solution.

1. A first example illustrates how network coarsening can reveal fundamental topological properties of a complex network, which may remain hidden in a full rendering of the full network.
2. A second study investigates how user-driven uncoarsening of specific regions of the n-reactome biochemical network can reveal hidden local structures with no need to iterate over the entire network.
3. A third study explores a gene-disease network to illustrate the possibility of switching between different perspectives by coarsening just one layer at a time, indicating that users can easily obtain relevant contextual information by on-demand navigation and exploration of the network.
4. A fourth study explores a scientific collaboration network (authors and papers) obtained from papers published related to COVID-19 and the “CoronaVac” vaccine. This study illustrates how users can explore groups in different contexts after obtaining a global visual perspective of how they are connected.

### 5.1. Revealing Hidden Patterns

The overlapping of graphical elements in node-link visualizations of large networks contributes to blurring important topological patterns. Consider, as an example, the bipartite network “HB/jagmesh7,” from the SuitSparse Matrix Collection.^4^ This network, with 1, 138 nodes and 3, 156 links, has a peculiar topology with three holes—a hole is a loop formed by nodes with cyclic links only and no links crossing the loop. Holes may indicate a relevant large-scale event occurring at a particular region of the network.

**Figure 4:**
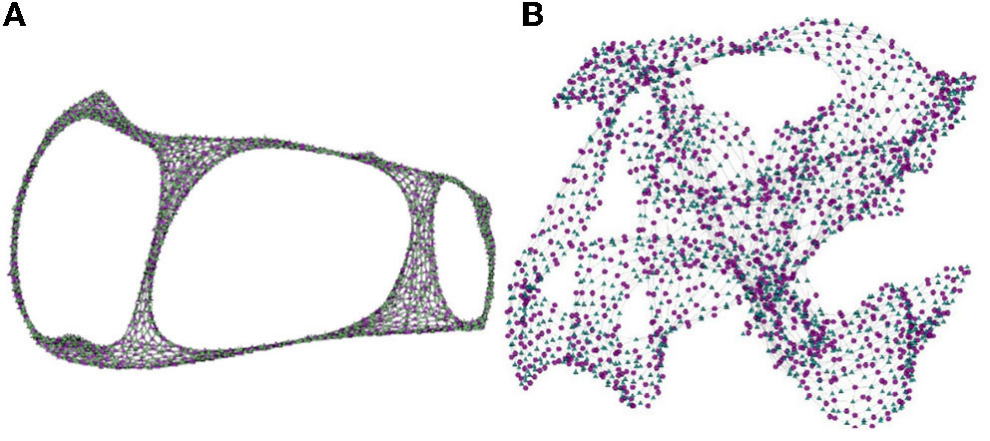
Bipartite network “HB/jagmesh7,” with $|V^{1}|=1,138$ and $|V^{2}|=1,138$ nodes, 7, 450 links and three holes: **(A)** a high-resolution rendering the network (from https://sparse.tamu.edu/HB/jagmesh7); **(B)** a rendering of the full network obtained in the BiNetViz system using the Fruchterman-Reingold layout algorithm, where the holes are not clearly visible.

Figure 4A, replicated from the SuitSparse Matrix site, illustrates a static high-resolution drawing of this network. In this large-sized high-resolution image the node layout algorithm had sufficient space to place the graphical elements so that the three holes are clearly visible. However, such rendering would hardly afford real-time user interaction. More commonly, users interact with node-link layouts displayed in limited-size screens. Figure 4B illustrates a rendering of the full “HB/jagmesh7” network obtained in BiNetVis with the Fruchterman-Reingold layout algorithm (Fruchterman and Reingold, 1991). In this view the hole topology of the network is not as clear. Even though it is possible to rotate a layout, or to create and render alternative layouts, they will not necessarily convey the existing holes, of which an analyst may not be aware. Moreover, a node-link screen rendering of a network this size also suffers from severe overlapping of graphical elements, which impairs legibility, interpretation, and interactivity.

**Figure 5:**
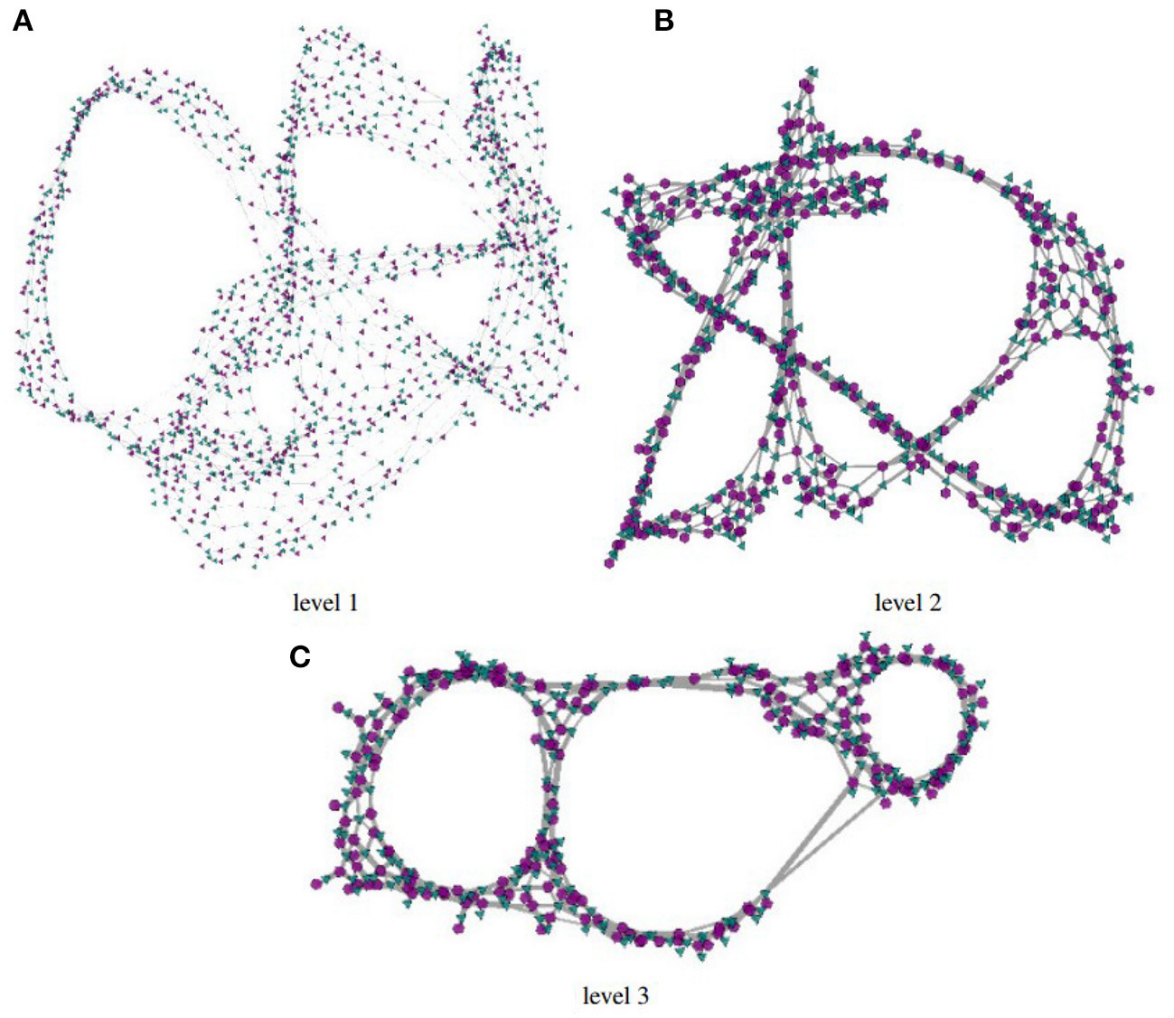
Node link layouts of the “HB/jagmesh7” network obtained in BiNetViz, at three increasing levels of coarsening: in **(A)** the network has $|V^{1}|=623$ and $|V^{2}|=626$ nodes and 2, 291 links; in **(B)** it has $|V^{1}|=342$ and $|V^{2}|=357$ nodes and 1, 289 links; whereas, in **(C)** it has $|V^{1}|=194$ and $|V^{2}|=199$ nodes and 699 links. Node-link layouts have been obtained with the Fruchterman-Reingold algorithm, and the coarsening hierarchy has been computed with the *GMb* algorithm.

Figure 5 shows coarsened versions of the original network obtained with the *GMb* algorithm using the parameters as following: three levels of coarsening and a reduction factor of 50% at both levels. Again, node-link layouts have been computed with the Fruchterman-Reingold algorithm. The coarsest network model shown in Figure 5C has just $|V^{1}|=194$ and $|V^{2}|=199$. Yet, the holes in the network topology remain evident in the three reduced models, and the layout in Figure 5C has a similar shape to the one shown in Figure 4A. In both the largest hole is placed in the central area, whereas the smallest hole appears to the right. Whilst detailed topological information is not accessible in the coarsened models, the renderings still preserve the essential global topological structure of the full network. Once the global structure is conveyed, a user may further investigate local topological structures by interactively selecting and expanding nodes.

### 5.2. On-Demand User Interaction

The interaction patterns between genes and proteins establish the basis of molecular biology and disease pathogenesis, i.e., the study of how diseases arise and evolve (Pawson and Linding, 2008; AlQuraishi et al., 2014). Protein groups are defined by the topological properties of the networks they entail, which allows for the isolation of functional and disease pathways (Sharan et al., 2007; Barabási et al., 2011). We illustrate a brief case study of BiNetViz on the n-reactome biochemical network, also available at the SuiteSparseMarix (https://sparse.tamu.edu/Schulthess/N_reactome).

**Figure 6:**
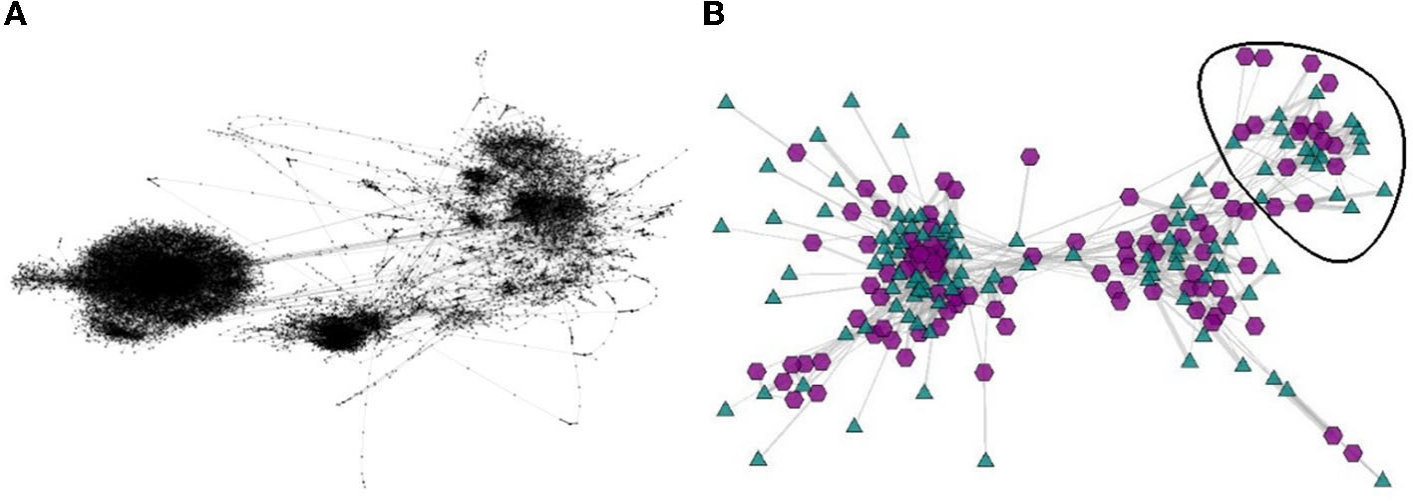
Two renderings of the n-reactome bipartite network with 24, 211 nodes (a layer with 8, 788 protein nodes and a layer with 15, 422 n-reactome interactions), and 41, 087 links. Panel **(A)** depicts a rendering of the full network, replicated from https://sparse.tamu.edu/Schulthess/N_reactome; panel **(B)** shows a rendering obtained in BiNetViz of a coarsened representation with only 200 nodes (100 in each layer). The protein nodes are represented as purple hexagons, whereas the reactome interactions are represented as green triangles.

Figure 6A depicts a high quality rendering of the original two-layer network, formed by 8, 788 proteins in one layer, 15, 422 reactome interactions in the second layer, and 41, 087 links between proteins and interactions. In this high-quality rendering one observes three more salient communities and several smaller ones. Figure 6B illustrates a coarsened representation obtained in BiNetViz, with 100 nodes in each layer (protein nodes represented as purple hexagons, and reactome interactions represented as green triangles). The coarser networks have been obtained executing three steps of algorithm *MLPb*, setting a 50% reduction factor at each coarsening level with an upper bound of 0.1 to allow for unbalanced super-nodes. Notice how the coarsest network, even after severe reduction, still mirrors the predominant characteristics of the original one, emphasizing the presence of three major communities and their connections.

**Figure 7:**
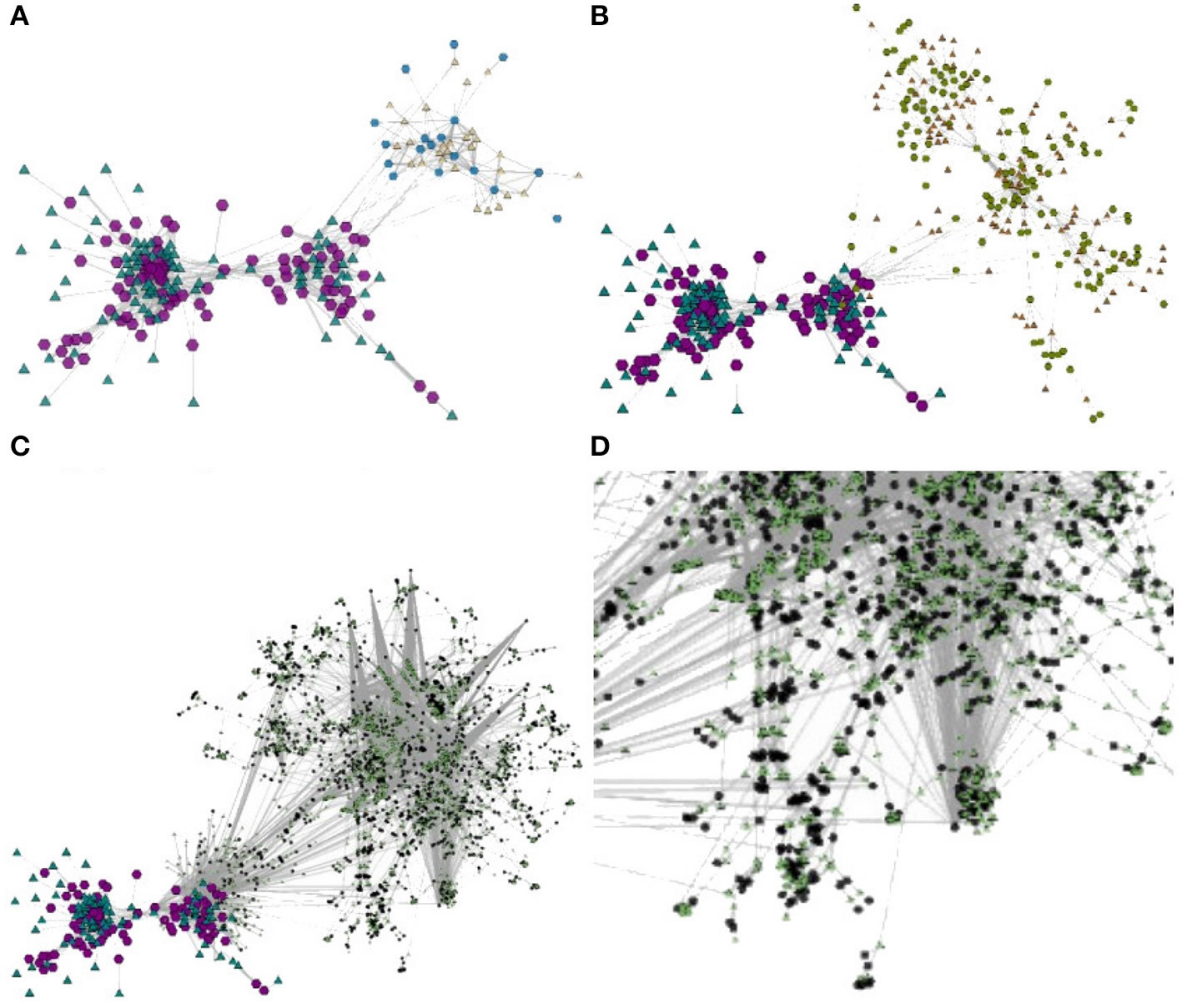
Departing from the coarsest network (level 3) depicted in Figure 6B, with just 100 nodes in each layer, one may select nodes for progressively more detailed visualizations. This is illustrated assuming a selection of the nodes enclosed within the black curve in Figure 6B. In **(A)** the selection is rendered at coarsening level 2; in **(B)** it is rendered at coarsening level 1; and in **(C)** it is rendered at level 0, equivalent to the original network. The non-selected nodes remain rendered at the coarsest level 3. Panel **(D)** shows a zoomed-in view of the drawing in **(C)**. These hybrid renderings, depicting the network at different levels-of-detail are created on-demand as a user interacts and selects sub-networks, triggering the corresponding uncoarsening operation. Selecting sub-networks to go back to coarser views is also possible.

For purposes of illustrating the possibilities afforded by BiNetViz we chose to inspect the connectivity node patterns of the smallest community, identified by the nodes within black circle depicted in Figure 6B. Figures 7A–C depict this particular community rendered, respectively, at coarsening levels 2, 1 and as in the original level, whereas the remaining nodes are still rendered at coarsened level 3. One observes how the complex topology is gradually unfolded in the sequence of views, each one revealing more detail.

A zoomed view of the community rendered at level 0 is depicted in Figure 7D, where one observes a group of densely connected nodes in the central area. As these nodes clearly correspond to numerous protein reactome interactions, they are likely to play a central role in the network, and an analyst might wish to further investigate the topological structures in that area. Or, she might become interested in examining the less connected nodes, in an attempt to understand their behavior or specific roles.

This example illustrates how BiNetVis could be useful for revealing localized patterns and topological relations involving groups of nodes from one or both layers of a large bipartite network. The framework allows focusing on particular areas for detail on the connections, preserving interactivity without loosing the global context provided by the remainder of the network. For this particular network, it could support, e.g., studying groups of proteins that could be targeted for therapeutic or other purposes, or groups of genes associated with metabolic deficiency.

### 5.3. Exploring a Gene-Disease Network

Goh et al. (2007) discuss how a network of human diseases and genes linked by known disease-gene associations offers a platform to investigating “whether human genetic disorders and the corresponding disease genes might be related to each other at a higher level of cellular and organism organization.” Authors use an available repository with data on diseases, genes and their associations to construct a bipartite network in which one layer has nodes corresponding to known genetic disorders, and the other layer has nodes corresponding to all known disease genes in the human genome. At the time, repository listed 1, 284 diseases and 1, 777 genes. A disease and a gene are connected with a link if mutations in the gene are implicated in the manifestation of the disease.

The authors manually classified each disease into one of 22 classes, based on the physiological system affected. They analyzed one-mode projections, i.e., derived unipartite models of the bipartite network, creating two projections: a human disease network, obtained connecting any two disease nodes that share at least one gene in common, i.e., gene mutations are associated to both diseases; a gene disease network connecting any two gene nodes connected to a common disease, i.e., known to be associated with the same disorder.

**Figure 8:**
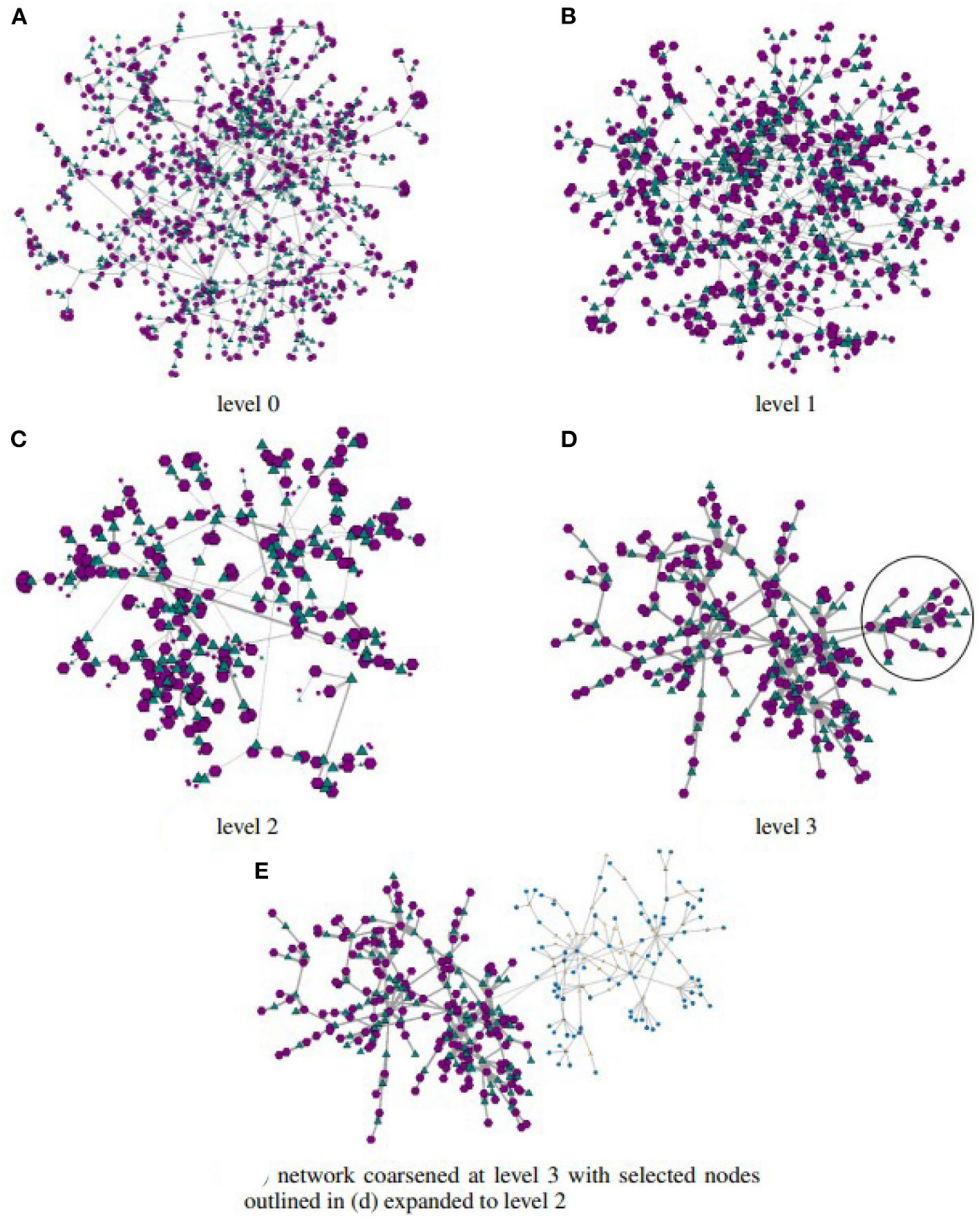
Biological bipartite network depicting the relationships between 516 diseases (purple hexagons) and 903 genes (green triangles). Panel **(A)** shows the initial network, which is depicted at increasing levels of coarsening in **(B)** with $|V^{1}|=323$ and $|V^{2}|=485$ super-nodes and 866 super links, **(C)** with $|V^{1}|=214$ and $|V^{2}|=273$ super-nodes and 524 super-links, and in **(D)** with $|V^{1}|=142$ and $|V^{2}|=161$ super-nodes and 328 super-links; finally panel **(E)** shows the expansion back to level 2 of the elements within the black circle in **(D)**, detailing an interesting topology.

BiNetViz offers an alternative approach for direct investigation of the bipartite network topology. Figure 8A illustrates a giant component of the original network formed by $|V^{1}|=516$ nodes representing diseases (depicted as purple hexagons) and $|V^{2}|=903$ nodes representing genes (depicted as green triangles), connected by 1, 550 links. Figures 8B–E illustrate this giant component at increasing levels of coarsening (levels 1, 2, and 3, respectively). The network was coarsened with the *GMb* algorithm, set with three coarsening levels, each level reduced by a factor of 50% relative to the previous one. Figure 8E illustrates the expansion of the super-nodes outlined with a black circle in Figure 8D to level 1 (expanded nodes depicted in blue and yellow representing genes and diseases, respectively). This feature enables a user to navigate through multiple levels-of-detail, e.g, getting an overview of the whole network and, at the same time, locally expanding (or contracting) selected regions for a more detailed observation. Therefore, multiple levels-of-detail may be rendered in a single node-link view.

Another interesting possibility afforded by BiNetViz is to conduct exploratory investigations by coarsening just one of the network layers at a time, possibly alternating the analysis focus between the gene and the disease layers. Based on this premise, the node-link view can display the layers at distinct granularity levels, whilst still preserving the full connectivity information between both layers. For instance, coarsening the disease layer, while keeping the gene layer at its original resolution, allows identifying groups of diseases associated with similar genes, which will become super nodes in the coarsened representation. This strategy allows identifying the genes related with similar diseases. Furthermore, a specific super-node representing a group of diseases can be uncoarsened to reveal their connectivity to a particular gene. Next we illustrate such a hypothetical scenario.

**Figure 9:**
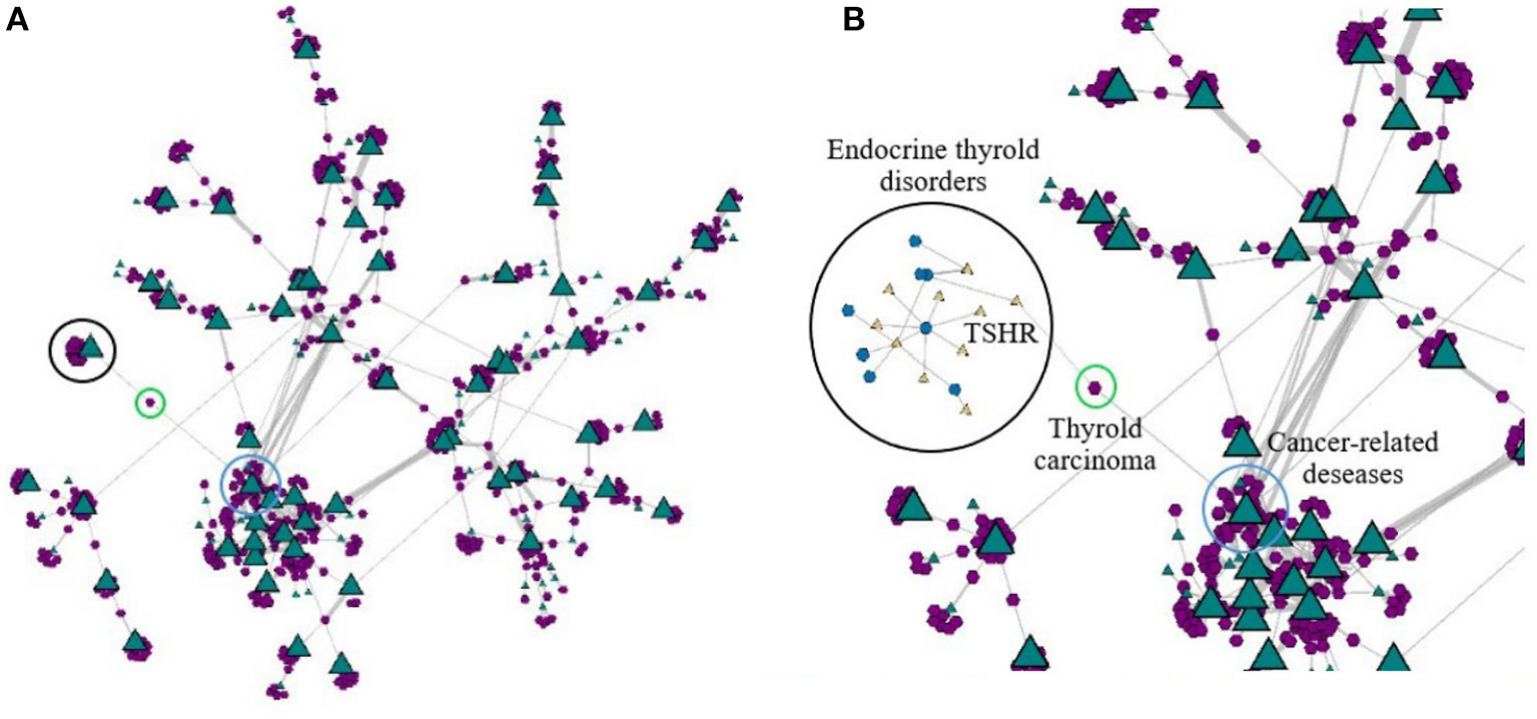
An exploratory investigation conducted over the network after coarsening the gene layer (represented by the green triangles) only: panel **(A)** depicts the coarsened gene layer; panel **(B)** depicts the local expansion of the super-nodes enclosed in the black circle in **(A)**.

Figure 9 illustrates a rendering of the giant component of the network, in which only the gene layer (green triangles) has been coarsened. Coarsening the gene layer allows exploring the network with a focus on the diseases and, if necessary, it is possible to expand the gene-layer locally on-demand by “exploding” super-nodes into their predecessor nodes. In this perspective, groups of diseases become more evident, which is convenient for detailed exploration.

Highly connected super-vertices thus represent densely connected groups of genes. Figure 9A depicts the giant component with the gene layer coarsened at level 4. The degree of the gene nodes is mapped to the triangle size, so that larger triangles indicate higher degrees. We notice how the disease nodes appear clustered around the gene super-nodes, e.g., the two gene super-nodes outlined by the light blue and the black circles are highly connected to several disease nodes. One may expand again those regions, for more detail. Figure 9B illustrates the local expansion of the gene super-node (green triangle) enclosed by the black circle. The expanded region includes diseases nodes (purple hexagons) related to “Endocrine” diseases, namely: “Hypothyroidism,” “Goiter,” “Thyroid hormone resistance,” “Total iodide organization defect,” “Graves disease” and “Autoimmune thyroid disease.” Interestingly, the disease highlighted with a green circle represents a type of cancer called “Thyroid carcinoma.” In this case, the TSHR-gene bridges the diseases Hyperthyroidism (Endocrine) and “Thyroid carcinoma” (Cancer). Notice that the “Thyroid carcinoma” is linked with another group of several cancer-related diseases (highlighted with a light blue circle), such as: “Oligodendroglioma,” “Multiple malignancy syndrome,” “Li-Fraumeni syndrome,” “Sezary syndrome,” “Lymphoma, “Adrenocortical carcinoma,” “Dermatobrosarcoma protuberans,” “T-cell lymphoblastic leukemia” and “Li-Fraumeni syndrome.” Future investigations could analyze whether the “Thyroid carcinoma,” as a cancer-related to the thyroid, could be explored as a bridge between cancer-related diseases and other thyroid diseases through the TSHR gene.

### 5.4. Exploring a Scientific Collaboration Network

We include an example illustrating how the BiNetViz approach can be applied to the analysis of scientific collaborations expressed in terms of co-authorship in papers. Such analysis can be aimed at assessing, for instance, scientific influence and scope of collaborations, influential groups of researchers on topics of interest, or the overall distribution structure of groups publishing on a particular topic.

The CORD-19 database is a freely available comprehensive collection of coronavirus literature accessible for data mining. Created as a joint effort by a coalition of renowned research organizations, the collection includes over 500,000 research papers about COVID-19, SARS-CoV-2, and similar coronaviruses, 200,000 of them with full text.^5^ We considered this database to create a bipartite network of papers and authors, with a focus on publications related with the “CoronaVac” vaccine developed by the Chinese pharmaceutical company *Sinovac Biotech*. Coronavac was one of the earliest vaccines adopted in Brazil, after an agreement for local production. We filtered the database to retrieve only papers published from 2020 for which the title or abstract included the term “CoronaVac” and created the bipartite network formed by the papers retrieved and their authors, linking the papers with their corresponding authors.

**Figure 10:**
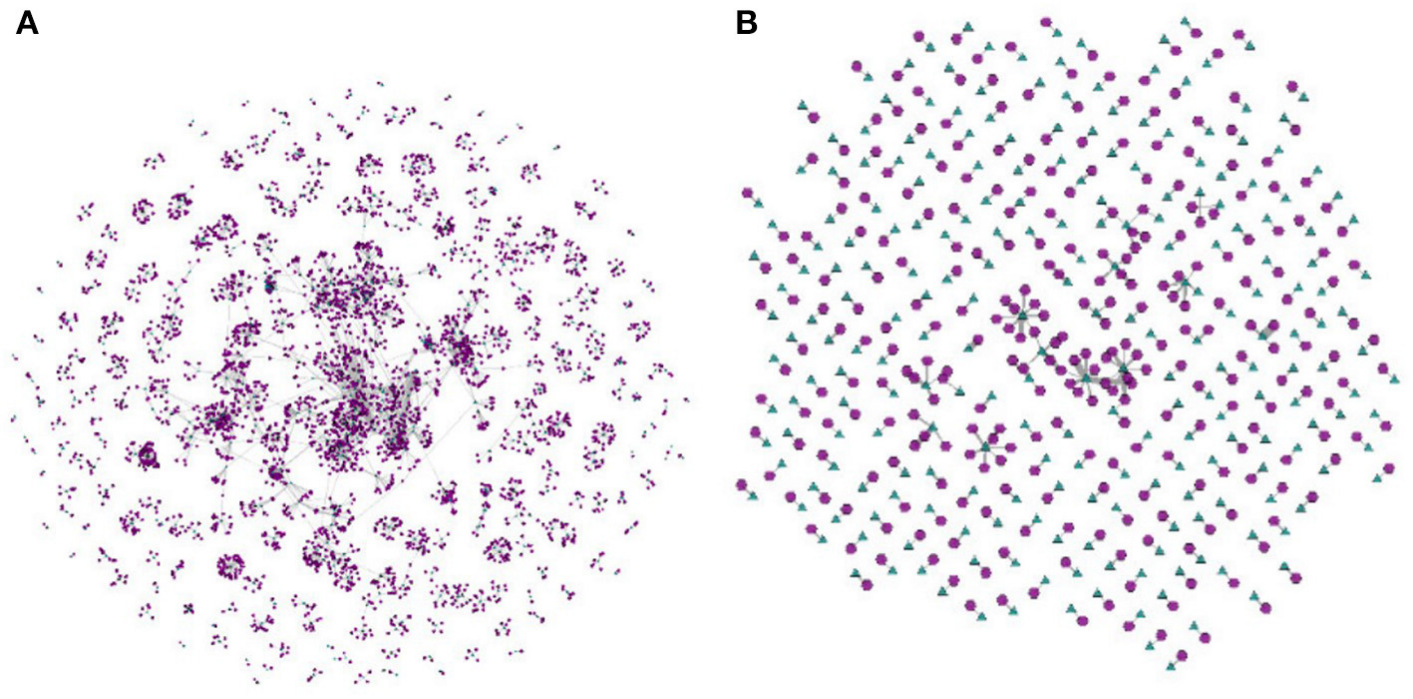
**(A)** Original scientific collaboration network formed by $|V^{1}|=3,531$ authors, $|V^{2}|=451$ papers and 5, 058 links. **(B)** the network coarsened at level 3, with $|V^{1}|=563$ author nodes, $|V^{2}|=264$ paper nodes, and 351 super-links. Purple hexagons depict author nodes or super-nodes, green triangles depict paper nodes or super-nodes.

The resulting network is formed by $|V^{1}|=3,531$ author nodes and $|V^{2}|=451$ paper nodes, with 5, 058 links indicating authorship. This network has been loaded in BiNetViz and it is displayed in Figure 10A with a node-link layout generated by the Fruchterman-Reingold algorithm. Author nodes are depicted as purple hexagons and paper nodes are depicted as green triangles. We notice many small isolated communities toward the boundary regions, and larger communities in the central area, apparently more connected, indicating group collaborations. A three-level hierarchy (levels 1 to 3) has been created applying algorithm *GMb* on both layers, with a target reduction factor at each level equal to 50%. The coarsened network at level 1 is formed by 1, 866 super-nodes in the author layer and 335 super-nodes in the paper layer; whereas for the networks at levels 2 and 3 these numbers are, respectively, 1, 119 and 563 author super-nodes and 286 and 264 paper super-nodes. Figure 10B depicts the coarsest network (level 3), in which the patterns of boundary isolated groups and central connected groups is again very evident. From this representation, users may easily select and expand communities of scientists and papers to obtain relevant contextual information by on-demand navigation and exploration.

**Figure 11:**
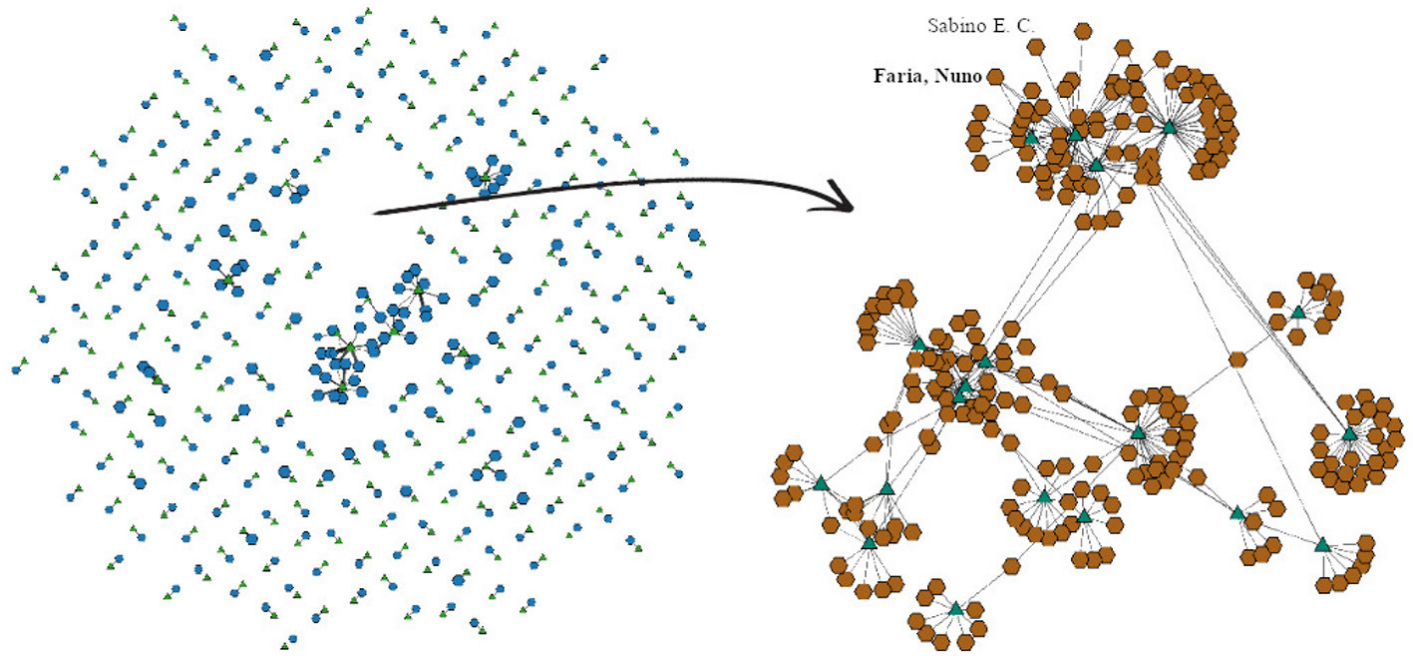
Further exploratory investigation of a selected group, departing from the coarsest network. The smaller central group in Figure 10B has been isolated and locally expanded back to level 0, for observation of its detailed topology.

**Figure 12:**
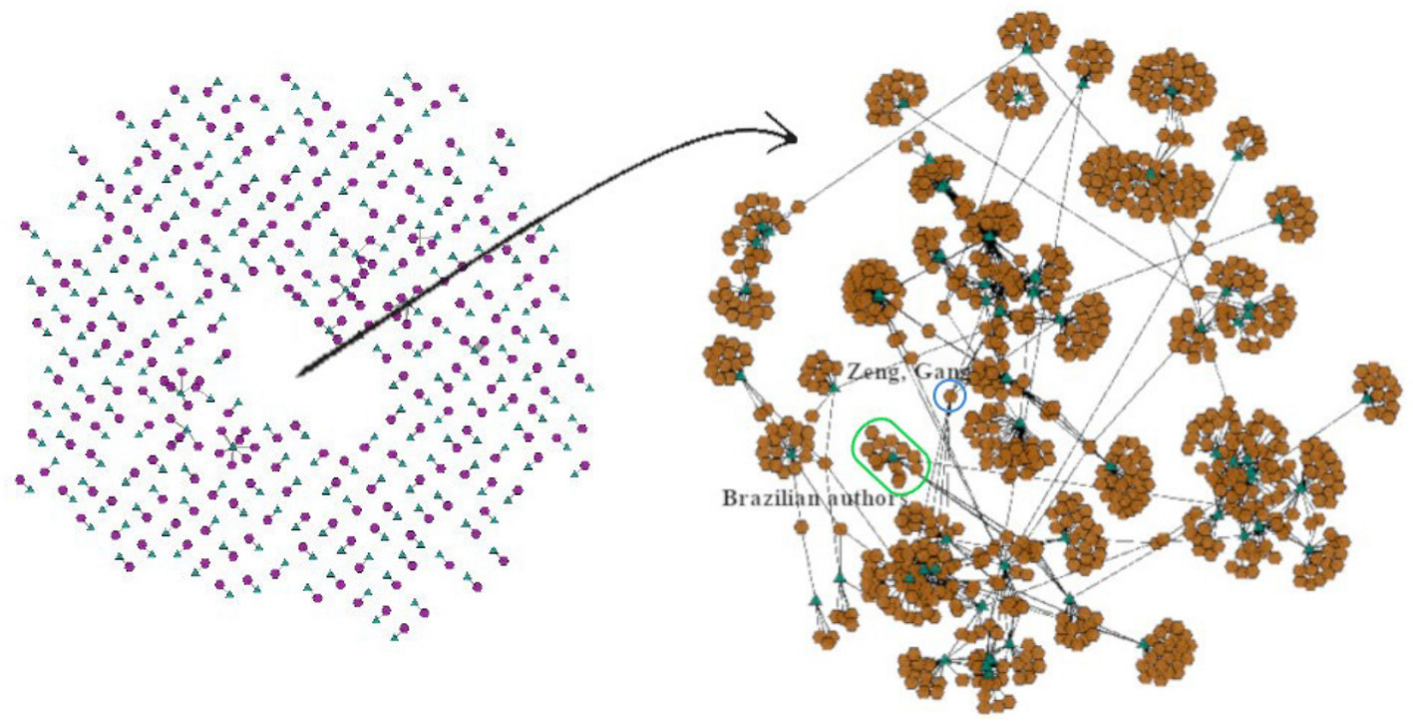
Further exploratory investigation of a selected group, departing from the coarsest network. The bigger central group in Figure 10B has been isolated and locally expanded back to level 0, for observation of its detailed topology.

Figures 11, 12 illustrate the expansion of two specific groups back to level 0 (brown octagons correspond to author super-nodes, whereas the green triangles depict paper super-nodes). A feature of BiNetViz enables to select and crop a group of elements, and then reposition it on the screen to navigate over multiple levels-of-detail. In this case the two groups identified in Figure 10B have been cropped from the main view and locally expanded. The smaller group is mostly related to Brazilian researchers, while the largest group is characterized by researchers related to the “Sinovac laboratory,” responsible by the “CoronaVac vaccine.” Figure 11 shows the expansion of the smaller group, which includes several sub-groups depicting teams led by reputed Brazilian researchers who contributed papers on the “CoronaVac” vaccine. For instance, Nuno Faria, a researcher affiliated with the University of Oxford, United Kingdom, has co-authored several studies related to the spreading of COVID-19 in Brazil, in partnership with Brazilian researchers, e.g., Li et al., 2022; Mee et al., 2022; Prete et al., 2022. This group also includes Ester C. Sabino (entry by “Sabino, E. C.”), immunologist and researcher at the University of São Paulo (USP). Several other visible groups and their connections could also be further investigated.

Figure 12 shows the expansion to level 0 of greater and denser group, which as in the previous case has been isolated prior to expansion. Important information can be identified in this sub-network, which corresponds to the largest group of connected authors and their papers. Highlighted within the blue circle, we notice a highly connected node corresponding to researcher “Zeng, Gang.” who is a medical director at Sinovac, the pharmaceutical company responsible for the development of the CoronaVac vaccine. The brown nodes indicated within the green line correspond to a small group of Brazilian researchers only, authors of the study represented in the green triangle visible in this group (Aikawa et al., 2022). This recently published paper reports results from a study on the immunogenicity pattern induced by the CoronaVac vaccine in SARS-CoV-2 seropositive patients with autoimmune rheumatic diseases. Interestingly, Ester C. Sabino is again one of the co-authors, however, now identified as “Sabino, Ester C.”. Of course, using the solution in this application domain would require additional processing, e.g., to identify replicate author entries. Again, many groups are visible in the detailed visualization that could be further investigated for connections and other information.

## 6. Conclusions and Further Work

We introduced a visualization framework for bipartite networks assisted by the multilevel method that admits a conceptual organization very consistent with the well-known visual information seeking mantra stated as *overview first, zoom and filter, then details-on-demand*. We introduce a system as a proof-of-concept on the feasibility of employing the multilevel strategy coupled with the familiar node-link views to visualize and investigate large bipartite networks. Our framework allows departing from an overview of the major topological structures in a network and then focus on relevant elements such as individual or multiple nodes, which may be selected and rendered at a less coarsened level, with further topological details displayed. The combination of the multilevel strategy with suitable software technologies and computationally inexpensive design decisions regarding the rendering of node-link representations yields a visual interface manageable with simple interactive operations that can effectively support navigation on large-scale networks.

The proposed visualization framework may be incorporated into interactive visualization systems of large networked data sets, aimed at different application domains, with added domain-specific functionalities for data analytics. It does require further validation on practical analytical settings, as the usage scenarios of interactive knowledge discovery are inherently complex, and often domain dependent. Indeed, approaches beyond those considered here could be devised for coarsening networks in general and bipartite networks in particular, e.g., coarsening could consider domain specific properties of the networked data. Further investigation on coarsening choices and their impact on executing exploratory visualization tasks in different application domains is required. As a downside, using this strategy does require from users some familiarity with the multilevel coarsening algorithms.

Beyond data exploration, we believe our current implementation could be tuned into a tool to guide and inform developers of novel multilevel algorithms and applications. For instance, certain multilevel strategies require selecting pivot nodes to guide the coarsening procedure, a task that could benefit from an interactive visual interface. The same applies to developers who wish to compare the outcome of multiple executions of coarsening algorithms, e.g., with different parameter settings. Finally, despite our current focus and interest on bipartite networks, a similar underlying rationale is clearly applicable to unipartite networks, or can be generalized to heterogeneous networks, as long as the underlying multilevel methods are provided to build a hierarchical representation.

## Data Availability Statement

Publicly available datasets were analyzed in this study. This data can be found here: https://sparse.tamu.edu/HB/jagmesh7, https://sparse.tamu.edu/Schulthess/N_reactome, and https://www.pnas.org/content/suppl/2007/05/03/0701361104.DC1.

## Author Contributions

AV, AA, and MO contributed the main conceptual ideas and underlying theory. AV, RF, and MO worked out the technical details in system development. All authors listed contributed in the literature review, planning of the case studies, analysis and interpretation of results, structuring, and writing the text. All authors read and revised the original draft and the final manuscript, and approved it for publication.

## Funding

This research was funded by the Coordenação de Aperfeiçoamento de Pessoal de Nível Superior—Brasil (CAPES)—Finance Code 001; the Center for Artificial Intelligence (C4AI-USP), with support by the São Paulo Research Foundation (FAPESP) and by the IBM Corporation under grant FAPESP 2019/07665-4; FAPESP grants 2017/05838-3, 2018/22214-6, 2013/07375-0, 2015/50122-0, and 2019/14429-5, and the Brazilian National Research Council (CNPq) research fellowships 304040/2019-3, 301847/2017-7 and 303199/2019-9.

## Conflict of Interest

The authors declare that the research was conducted in the absence of any commercial or financial relationships that could be construed as a potential conflict of interest.

## Publisher's Note

All claims expressed in this article are solely those of the authors and do not necessarily represent those of their affiliated organizations, or those of the publisher, the editors and the reviewers. Any product that may be evaluated in this article, or claim that may be made by its manufacturer, is not guaranteed or endorsed by the publisher.

## Footnotes

^1^By “large-scale” we mean networks with up to a few hundreds of thousands nodes, in contrast to massive-scale networks with millions to billions nodes.

^2^source code and installation instructions at https://github.com/ttm/ml-net-gui (runs on Linux platforms).

^3^ http://lgl.sourceforge.net/#FileFormat

^4^ https://sparse.tamu.edu/

^5^ https://www.kaggle.com/datasets/allen-institute-for-ai/CORD-19-research-challenge
